# Derivation and Validation of a Screening Model for Hypertrophic Cardiomyopathy Based on Electrocardiogram Features

**DOI:** 10.3389/fcvm.2022.889523

**Published:** 2022-05-24

**Authors:** Lanyan Guo, Chao Gao, Weiping Yang, Zhiling Ma, Mengyao Zhou, Jianzheng Liu, Hong Shao, Bo Wang, Guangyu Hu, Hang Zhao, Ling Zhang, Xiong Guo, Chong Huang, Zhe Cui, Dandan Song, Fangfang Sun, Liwen Liu, Fuyang Zhang, Ling Tao

**Affiliations:** ^1^Department of Cardiology, Xijing Hospital, The Fourth Military Medical University, Xi’an, China; ^2^Department of Ultrasound, Xijing Hospital, The Fourth Military Medical University, Xi’an, China

**Keywords:** electrocardiogram (ECG), screening model, hypertrophic cardiomyopathy, left ventricular hypertrophy, C-statistic

## Abstract

**Background:**

Hypertrophic cardiomyopathy (HCM) is a widely distributed, but clinically heterogeneous genetic heart disease, affects approximately 20 million people worldwide. Nowadays, HCM is treatable with the advancement of medical interventions. However, due to occult clinical presentations and a lack of easy, inexpensive, and widely popularized screening approaches in the general population, 80–90% HCM patients are not clinically identifiable, which brings certain safety hazards could have been prevented. The majority HCM patients showed abnormal and diverse electrocardiogram (ECG) presentations, it is unclear which ECG parameters are the most efficient for HCM screening.

**Objective:**

We aimed to develop a pragmatic prediction model based on the most common ECG features to screen for HCM.

**Methods:**

Between April 1st and September 30th, 2020, 423 consecutive subjects from the International Cooperation Center for Hypertrophic Cardiomyopathy of Xijing Hospital [172 HCM patients, 251 participants without left ventricular hypertrophy (non-HCM)] were prospectively included in the training cohort. Between January 4th and February 30th, 2021, 163 participants from the same center were included in the temporal internal validation cohort (62 HCM patients, 101 non-HCM participants). External validation was performed using retrospectively collected ECG data from Xijing Hospital (3,232 HCM ECG samples from January 1st, 2000, to March 31st, 2020; 95,184 non-HCM ECG samples from January 1st to December 31st, 2020). The C-statistic was used to measure the discriminative ability of the model.

**Results:**

Among 30 ECG features examined, all except abnormal Q wave significantly differed between the HCM patients and non-HCM comparators. After several independent feature selection approaches and model evaluation, we included only two ECG features, T wave inversion (TWI) and the amplitude of S wave in lead V1 (SV1), in the HCM prediction model. The model showed a clearly useful discriminative performance (C-statistic > 0.75) in the training [C-statistic 0.857 (0.818–0.896)], and temporal validation cohorts [C-statistic 0.871 (0.812–0.930)]. In the external validation cohort, the C-statistic of the model was 0.833 [0.825–0.841]. A browser-based calculator was generated accordingly.

**Conclusion:**

The pragmatic model established using only TWI and SV1 may be helpful for predicting the probability of HCM and shows promise for use in population-based HCM screening.

## Highlights

### What Are the Novel Findings of This Work?

– Using multiple independent statistical approaches, the two easily acquired ECG parameters, TWI and SV1, were selected and showed satisfactory C-statistics of 0.871 and 0.833 validated by internal and external cohorts, respectively.
– We developed a pragmatic prediction model based on TWI and SV1, which can automatically be acquired by electrocardiography, to screen for HCM. The corresponding web-based calculator may be helpful for improving the detection rate of HCM in the general population.

## Introduction

Hypertrophic cardiomyopathy (HCM), one of the most common genetic cardiovascular diseases that is heterogeneous in its clinical profile and natural history including progressive heart failure (HF), atrial fibrillation (AF)/embolic stroke, and is frequently visible non–trauma-related sudden death in young asymptomatic student-athletes, which accounting for approximately one-third of these catastrophic events (1–5). It should also be recognized that approximately 60% of sudden deaths due to HCM occur in individuals during routine physical activity and not exclusively in athletes (6–8). The advancement of effective treatment interventions has significantly reduced the disease-related mortality rate to 0.5% (9–11). HCM is treatable and consistent with normal longevity; thus, the ability to diagnose HCM in a timely and convenient manner for detailed clinical assessment has increased substantially in importance. It is estimated that there are approximately 20 million HCM patients worldwide. However, 80–90% of HCM patients are still clinically unidentified, such a high rate of underdiagnosis may lead to an increased risk of HF, thromboembolic events attributable to AF, and sudden cardiac death (SCD) among these “hidden” patients, seriously endangering human health and social development (2, 5).

The current diagnostic criteria mainly rely on echocardiography (Echo) or cardiac magnetic resonance (CMR), combined with genetic testing, which contributed to substantial advancements in understanding this disease and facilitated patient management (12). However, these approaches are relatively unpopular and expensive, need expertized interpretation, and have high inter- and intrareader variability and lack defined conclusions of variant results, all of which have hampered the detection rate of HCM (13–15). Furthermore, since Asians have smaller left ventricular volumes than Caucasians, the diagnostic threshold of maximum wall thickness (MWT) for Asians should be reduced accordingly (recommended from 15 mm to 10–12 mm) (16). This will then further increase the prevalence and underdiagnosed rate of HCM in Asia, as well as the incidence of potential adverse events, including in China.

More than 90% of HCM patients have abnormalities on electrocardiogram (ECG), which may be the only early manifestation of HCM (17–19). Compared to imaging modalities such as echocardiography and cardiac magnetic resonance (CMR), ECG is more convenient, non-invasive, less expensive, and potentially more sensitive for detecting left ventricular hypertrophy (LVH) in the context of HCM screening. Current guidelines recommend 12-lead ECG for the initial clinical evaluation of patients with HCM (2, 20). With a vast array of ECG abnormalities, there are no simple, convenient and pragmatic models to use in screening for HCM. Furthermore, there is a lack of specialized ECG interpreters, especially in underdeveloped countries and regions.

In the current study, we aimed to develop a practical model based on the most common and easily acquired ECG features by electrocardiography as an initial screening tool for HCM to improve the detection rate of HCM in the population, prevent adverse cardiac events, and improve long-term prognosis.

## Materials and Methods

### Study Population

**FIGURE 1:**
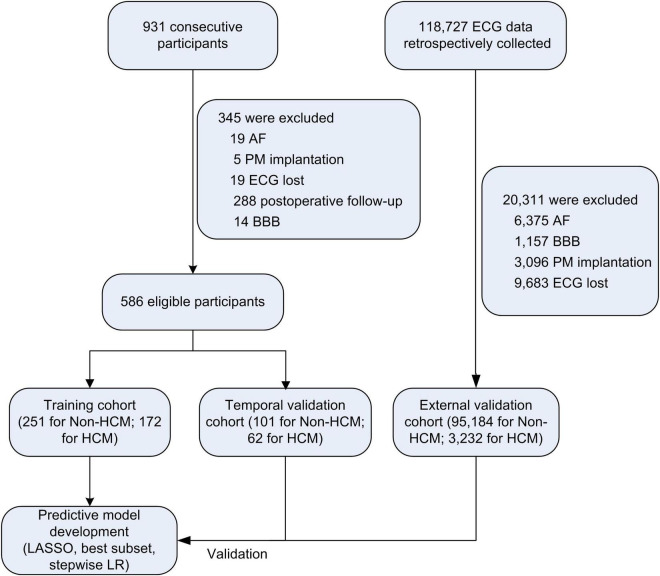
Study flow chart. AF, atrial fibrillation; PM, pacemaker; BBB, bundle branch block; HCM, hypertrophic cardiomyopathy; LASSO, the least absolute shrinkage and selection operator; LR, logistic regression.

Between April 1st and September 30th, 2020, 423 consecutive subjects from the International Cooperation Center for Hypertrophic Cardiomyopathy of Xijing Hospital [172 HCM patients, 251 participants without LVH (non-HCM)] were prospectively included in the training cohort, and between January 4th and February 30th, 2021, 163 participants from the same center were included in the temporal internal validation cohort (62 HCM patients, 101 non-HCM participants). External validation was performed using retrospectively collected ECG data from Xijing Hospital (3,232 HCM ECG datasets from January 1st, 2000, to March 31st, 2020; 95,184 non-HCM ECG datasets from January 1st to Dec 31st 2020). The study flowchart is shown in Figure 1.

HCM was diagnosed according to the European Society of Cardiology (ESC) guidelines for HCM (2, 12), which is defined as a maximum wall thickness (MWT) ≥ 13 mm or ≥ 15 mm for individuals with and without a family history of HCM, respectively, with the absence of any abnormal secondary causes, such as uncontrolled hypertension or aortic stenosis (AS), capable of producing such a magnitude of hypertrophy. Otherwise, classified as non-HCM. Patients who had previously been treated with an interventricular reduction procedure, including septal myectomy, alcohol septal ablation, and percutaneous intramyocardial septal radiofrequency ablation (21), or had a pacemaker with ventricular pacing, atrial fibrillation (AF), bundle branch block (BBB) and missing ECG data were excluded.

All enrolled participants had data from at least one standard 12-lead ECG and transthoracic cardiac echocardiography examination. The research protocol was approved by the ethics committee of Xijing Hospital, and the requirement for written informed consent for this analysis was waived by the institutional review board. The study was performed in accordance with local law and the regulations of Xijing Hospital and complied with the Declaration of Helsinki.

### Echocardiography Acquisition

Transthoracic standard two-dimensional and Doppler echocardiography measurements were obtained according to the recommendations of the American Society of Echocardiography and the European Association of Cardiac Imaging for cardiac chamber quantification by echocardiography in adults (22). The MWT was defined as the greatest thickness in any single segment (12, 23). The Doppler signals were collected from the mitral inflow, and the peak velocity of early E- and late A-waves was recorded at a speed of 100 mm/s (24).

### Electrocardiogram Evaluation

Routine 12-lead ECG for each eligible participant was acquired in the supine position at a sampling rate of 500 Hz, an amplitude of 10 mm/mv and a speed of 25 mm/s. The ECG features were measured automatically by the computer, and these data were checked independently by two experienced ECG reviewers blinded to clinical details. A total of 30 common ECG parameters were acquired. The mean heart rate (HR), P width, and PR and QT intervals were calculated by using three consecutive cycles. The QT interval was defined as the distance between the start of the QRS complex and the last point at which the T wave intersected the isoelectric line. The corrected QT (QTc) interval was calculated by using the Bazett formula. An abnormal Q wave was defined (25) as a Q wave with a width ≥ 3 mm or depth 1/4 of the ensuing R wave in two or more contiguous leads (except lead III and aVR). T wave inversion (TWI) (26) was diagnosed as an inverted T wave amplitude greater than 1 mm in two or more contiguous leads (excluding III, aVR, and V1). The amplitudes of the R and S waves were measured in all precordial leads and limb leads I, III and aVL. The presence of a pathologically high LV wall voltage was assessed by the amplitudes of RV5 + SV1 (RV5SV1, Sokolov–Lyon index), RaVL + SV3 (RaVLSV3, Cornell index), and RI + SIII (RISIII) (27). The amplitudes of the R and S waves in leads V1-4, reflecting interventricular septum (IVS) hypertrophy, were also included.

### Statistical Analysis

The sample size was determined by acquiring all available data during the investigation period. Since there was no formal statistical hypothesis of the study, no power calculation was done in advance. However, the minimum acquire sample size was determined. The data used for model development and validation has no missing values. Categorical variables are presented as the frequency and percentage, while normally distributed continuous variables are expressed as the mean and standard deviation (*SD*); otherwise, are expressed as the median and interquartile range (IQR). The baseline characteristics and ECG parameters were compared with *t*-tests or non-parametric Mann–Whitney *U*-tests, as appropriate (for continuous variables), and Fisher’s exact tests (for categorical variables).

Model development was performed according to the Transparent Reporting of a Multivariable Prediction Model for Individual Prognosis or Diagnosis (TRIPOD) guidance (Supplementary Table 1) (28). Four different approaches were applied in JMP Pro 16.0 for feature selection and model development: (1) the adaptive least absolute shrinkage and selection operator (LASSO) analysis; (2) LASSO followed by multivariable logistic regression with backward stepwise selection; (3) LASSO followed by best subset selection; and (4) multivariable logistic regression with backward stepwise selection. The comparison of Receiver operating characteristic (ROC) curves for different models with distinct variables were performed using the DeLong test (R package “pROC”) in the training and validation cohorts. The discriminative accuracy was quantified using the C-statistic. According to previous literature, a C-statistic less than 0.6 was considered to reflect poor discrimination; 0.60–0.75, possibly helpful discrimination; and greater than 0.75, clearly useful discrimination (29). The R packages ‘‘CalibrationCurves’’ and ‘‘ResourceSelection’’ were used to generate the calibration plots, and the Hosmer--Lemeshow test was used to assess the goodness-of-fit of the model. A browser-based calculator was generated accordingly.^1^

Statistical analyses were carried out using R software, version 4.1.1, JMP Pro 16.0 and SPSS 26.0. A two-tailed *P*-value < 0.05 was considered statistically significant.

## Results

### Baseline Characteristics

Baseline characteristics are presented in Table 1. The median age was 47 years, and there was no difference between the HCM and non-HCM groups (*P* > 0.05). In both the training and temporal internal validation cohorts, the male sex, hypertension and coronary artery disease were more common in HCM patients compared with non-HCM participants (all *P* < 0.05). Although there was no difference in the left ventricular ejection fraction (LVEF) between the HCM and non-HCM groups (*P* > 0.05), the average MWT (22 mm vs. 8 mm) and left atrial diameter (43 mm vs. 35 mm) were larger in the HCM group than in the non-HCM group, as was the incidence of LV diastolic dysfunction, reflected by E/A (all *P* < 0.05).

**TABLE 1:** Baseline characteristics.

	Overall			Training cohort			Temporal validation cohort		
			
	non-HCM	HCM	*P*	non-HCM	HCM	*P*	non-HCM	HCM	*P*
								
	*N* = 352	*N* = 234		*N* = 251	*N* = 172		*N* = 101	*N* = 62	
Age, y (mean *SD*)	47 (16)	47 (15)	0.584	47 (16)	47 (15)	0.670	45 (15)	46 (15)	0.799
Male	191 (54.3)	163 (69.7)	<0.001	137 (54.6)	120 (69.8)	<0.001	54 (53.47)	43 (69.35)	0.045
**Co-existing conditions**									
Hypertension	65 (18.47)	91 (38.89)	<0.001	49 (19.52)	73 (42.44)	<0.001	16 (15.84)	18 (29.03)	0.044
CAD	34 (9.66)	40 (17.09)	0.008	29 (11.55)	38 (22.09)	0.004	5 (4.95)	2 (3.23)	0.710
CA	36 (10.23)	0 (0)	<0.001	22 (8.76)	0 (0)	0.008	14 (13.86)	0 (0)	0.001
AS	14 (3.98)	0 (0)	0.002	10 (3.98)	0 (0)	<0.001	4 (3.96)	0 (0)	0.299
**Echocardiography parameters**									
MWT, mm	9 (8, 11)	22 (18, 26)	<0.001	9 (8, 11)	22 (18, 26)	<0.001	10 (8, 12)	21 (18, 26)	<0.001
LA, mm	35 (33, 37)	43 (38, 46)	<0.001	35 (33, 37)	43 (38, 46)	<0.001	34 (32, 36)	43 (39, 46)	<0.001
LVEF,%	60 (56, 63)	59 (56, 62)	0.409	60 (57, 63)	59 (56, 62)	0.458	59 (56, 63)	59 (57, 61)	0.725
E/A < 1	119 (33.8)	193 (82.5)	<0.001	75 (29.9)	154 (89.53)	<0.001	44 (43.6)	39 (62.90)	0.016
SAM sign	0 (0)	122 (52.14)	<0.001	0 (0)	96 (55.81)	<0.001	0 (0)	26 (41.94)	<0.001

*Data are expressed as n (%) or median (IQR), unless otherwise specified.*

*IQR, inter-quartile range; CAD, coronary artery disease; CA, cardiac amyloidosis; AS, aortic stenosis. MWT, maximum wall thickness; LA, left atrium; LVEF, left ventricular ejection fraction; E/A, E/A ratio, mitral peak E/A wave velocity ratio; SAM, systolic anterior motion.*

### Electrocardiogram Characteristics

Except for abnormal Q wave, all remaining 29 ECG variables showed a significant difference between the HCM and non-HCM groups. HCM patients had a longer P wave, QRS interval, and QTc interval and higher R and S wave amplitudes in all precordial leads. HCM patients also had higher amplitudes in limb leads I and III (all *P* < 0.05). Parameters of hypertrophy, such as RISIII, RV5SV1, and RaVLSV3, other parameters reflecting interventricular hypertrophy, including the R or S wave amplitude in lead V1, V2, and V3, and combinations of RV1V2, SV1V2, RV2V3, SV2V3, RV3V4, and SV3V4, were higher in the HCM group than in the non-HCM group. In addition, TWI was more common in the HCM than in the non-HCM group, both in the training and temporal internal validation cohorts, respectively, all *P* < 0.01) (Supplementary Table 2).

### Selection of Predictors and Construction of Prediction Model for Hypertrophic Cardiomyopathy

ECG variables, age, and sex were included for variable selection. Four different approaches were used for feature selection, including the adaptive LASSO analysis (Supplementary Table 3), multivariable logistic regression with backward stepwise selection (Supplementary Table 4), LASSO followed by multivariable logistic regression with backward stepwise selection (Supplementary Table 5), and LASSO followed by best subset selection (Supplementary Table 6). Several indexes were calculated, including the Akaike information criterion (AIC), Bayesian information criterion (BIC), R^2^ and C-statistic of each model constructed by different combinations containing a distinct number of ECG variables.

To evaluate the performance of each model, we focused on the AIC, BIC, R^2^, and C-statistic. In general, the model with the smallest AIC is preferred. However, we found that in the temporal internal validation cohort, models with 2 variables (TWI + SV1; TWI + RV5SV1) had C-statistics of 0.871 and 0.872, R^2^ of 0.354 and 0.323, but with the biggest AIC (449.023 vs. 393.220) and BIC (461.108 vs. 405.305). Furthermore, the model constructed by TWI and SV1 had a smaller AIC and BIC compared with the one constituted by TWI and RV5SV1. When the number of variables ranged from 9 to 13, the C-statistics of the models ranged from 0.923 to 0.939, but with the relatively small AIC (ranging from 322.709 to 310.423) and BIC (374.435–350.363) values. Considering the moderate decrease in the C-statistics from the models with the smallest AIC values to the models with 2 variables (Supplementary Figure 1) and the credendum that models with fewer variables have greater clinical applicability, we selected the simplest model with 2 variables as having the best performance for HCM screening. The comparison using Delong test of 2 models with TWI + SV1 and TWI + RV5SV1 was shown in Supplementary Table 7 and Supplementary Figure 2, indicating no significant difference between the two models, even the former showed slightly better performance in the external validation cohort. Finally, the TWI and SV1 was selected to construct the HCM model, the following formula shown as:


Y=-2.714+2.146×TWI+1.337×SV1(TWI=1,NoTWI=0).

### Estimation of Discriminative Performance for Hypertrophic Cardiomyopathy Screening Model

**FIGURE 2:**
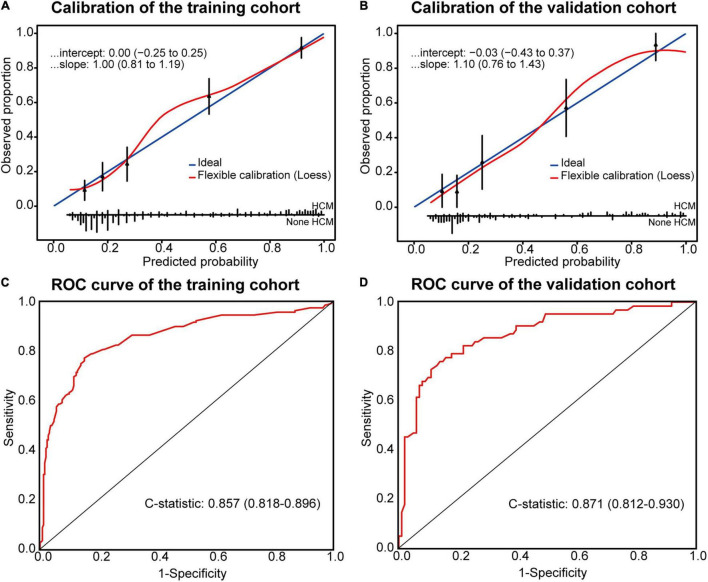
Calibration plots and ROC curve for the Model. Calibration plots between predicted and observed HCM in the training **(A)** and temporal validation **(B)** cohorts. The 45° blue line represents a perfect prediction, and the red line represents the predictive performance of the model. ROC curve of the model in the training **(C)** and temporal validation **(D)** cohorts.

The Hosmer–Lemeshow test showed χ^2^ as 10.037 (*P* = 0.262) in the training and 7.714 (*P* = 0.462) in the temporal validation cohort, indicating good fitness of the model. Calibration plots showed a good imitative effect of the model, with slope (1.00 vs. 1.10) and intercept (0.00 vs. -0.03) in the training and temporal validation cohorts, respectively (Figures 2A,B). The C-statistics of the model were 0.857 (0.818–0.896) in the training cohort, and 0.871 (0.812–0.930) in the temporal internal validation cohort (Figures 2C,D).

**FIGURE 3:**
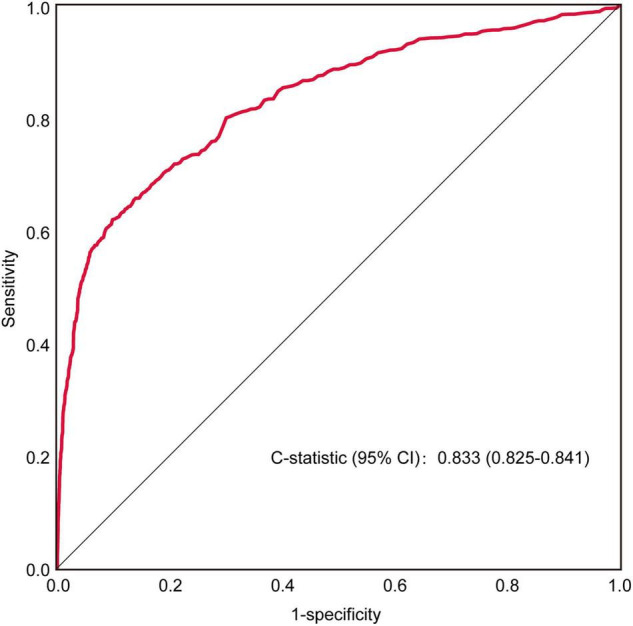
ROC curve for the model in the external validation cohort.

Furthermore, receiver operating characteristic (ROC) curve analysis of retrospectively collected data from a large-population-based, external validation cohort (HCM = 3,232 vs. non-HCM = 95,184) yielded a C-statistic of 0.833 (0.825–0.841) (Figure 3). When the false-negative rate was 10%, the false-positive rate was 54%.

### Examples Illustration

**FIGURE 4:**
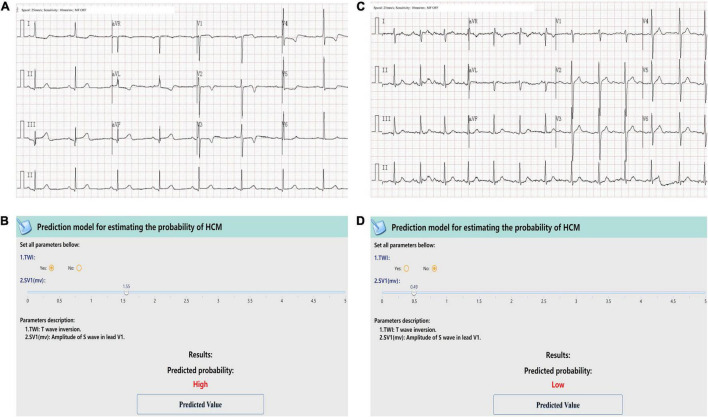
Examples of the screen-shots of the web-based calculator. The first case was a 57-year-old male. ECG showed sinus rhythm, SV1 = 1.55 mV, and TWI in precordial leads V2 to V4. ECG showed no indications of LVH **(A)**. A screenshot of the web-based calculator indicated a high probability of HCM and recommended a referral to a dedicated HCM center. Then the patient was diagnosed as HCM, with an MWT of 23 mm and an LVOTG of 50 mmHg at rest by Echo **(B)**. The second case was a 35-year-female. Her ECG showed sinus rhythm with SV1 of 0.49 mV and no TWI **(C)**. She was classified as having a low probability of HCM by the model **(D)**. The echocardiography found no obvious abnormality, ruling out HCM.

At last, the web-calculator was developed for clinical application (see text footnote 1). Figure 4 showed two case scenarios. The case one was a 57-year-old male with no evident discomforts. During an accidental examination in clinic, the ECG showed sinus rhythm, with P interval 120 ms, SV1 1.55 mV, and TWI in precordial leads V2 to V4, and no indications of LVH (Figure 4A). The online calculator indicated that the patient had a high probability of HCM (Figure 4B), so he was recommended a referral to our HCM center, and diagnosed as HCM, with an MWT of 23 mm and LV outflow tract gradient (LVOTG) of 50 mmHg at rest by echocardiography. The Case Two was a 35-year-old female. Her ECG showed sinus rhythm with SV1 of 0.49 mV and no TWI (Figure 4C). She was classified as having a low probability of HCM by the model (Figure 4D). Finally, she was diagnosed as systemic lupus erythematosus, and the echocardiography found no obvious abnormality, ruling out HCM.

## Discussion

In this study, we developed a screening model for HCM using the two most common and easily acquired ECG parameters and validated the model in a large-scale external validation cohort.

HCM is a major cause of SCD, HF and AF. Most HCM patients present with abnormalities on ECG. However, the abnormal ECG presentations in HCM are diverse, none are specific for HCM, and there are no standard and available models that can be used to quickly screen for HCM. Recently, an artificial intelligence (AI)-enabled ECG model using a convolutional neural network (CNN) also showed high sensitivity and accuracy for detecting HCM (30). Nevertheless, the precise features that the web sees are obscure and unexplainable through the AI process, and an advanced infrastructure may be required for its application. In the current study, TWI and SV1 were selected to identify HCM. These two variables can be automatically calculated and acquired by electrocardiography and might be more readily used to distinguish between HCM and non-HCM in a screening scenario.

Previous reports have suggested that repolarization abnormalities, such as TWI, are more indicative of HCM (31). TWI, especially in inferior and lateral leads, have been reported to account for nearly 70% of HCM cases (17). In the current study, we found TWI in nearly 60% of HCM cases and only 10% of non-HCM cases. It has been reported that among patients with giant TWI but a normal LV thickness on echocardiography, over 20% would exhibit progression and fulfill the criteria of HCM over a median follow-up period of 2 years (32). Therefore, TWI may be an early presentation of HCM. TWI has also been reported to be an indicator of the SCD risk in HCM. The ventricular repolarization dispersion due to ionic remodeling in coexisting regions of septal and apical hypertrophy may explain the normal QRS complex and TWI observed in the phenotype associated with an increased SCD risk score (33, 34).

A recent study (35) found that while 96% of HCM patients had abnormalities on ECG, only a few of the QRS voltage elevations reached the standard for LVH (RV5SV1). Among several LVH indexes, the Cornell index (RaVLSV3) has shown the greatest net sensitivity and specificity for diagnosing LVH in young HCM patients (27, 36). In the present study, indexes reflecting LVH and IVS hypertrophy, including RV5SV1, RaVLSV3, RISIII, RV2V3, SV2V3, RV3V4, and SV3V4 were significantly increased in HCM patients. However, compared to TWI and SV1, these indexes are less able to discriminate HCM from non-HCM (Supplementary Figure 3). With increasing hypertrophy or enlargement of the left ventricle, the effects of LVH become more prominent, resulting in an increase in the R wave amplitude in leads facing the left ventricle (leads I, aVL, V5 and V6) and a deepening of the S wave in leads facing the right ventricle or deviating from the left ventricle (V1 and V2). IVS hypertrophy in HCM changes the direction of septal depolarization from right-to-left to left-to-right; thus, the vector turns to the right and forward. Therefore, compared with non-HCM, in HCM, the S wave increases in V1 and V2. The systolic anterior motion (SAM) sign of obstructive HCM makes this trend more obvious, with greater increases in SV1 than SV2. This explains why SV1 is given more weight in the model for HCM screening.

HCM has become a highly treatable condition with effective options that alter natural progression along specific adverse pathways at all ages. ECG, with the advantages of wide availability, low-cost and high reproducibility, remains a mainstay in HCM management. The prediction model based on ECG might be more practical than the screening approaches such as Echo, CMR, or gene tests. Compared with the patent AI algorithm for HCM screening, the current formula and the online-calculator of the prediction model is open and free available. We believe that this prediction model may facilitate HCM screening in general population, especially in the undeveloped regions. Furthermore, the clinical significance of the screening model would be strengthened if the results of a cost-effectiveness analyses could be provided. We shall collect relative data and perform such analysis in our further study.

### Limitations

First, all the ECG data in the current study were from a single hospital. Further external validation using participants from multiple centers and a population with more heterogeneity is needed. Second, the analysis of ECG variables in the current study focused only on those parameters that are easily assessed in clinical practice; thus, some important but less frequently used variables might have been omitted. Third, we did not consider the effects of genotype on the ECG results, but some researchers, using an ECG-AI model, have reported that the result was correlated with the HCM phenotype rather than the genotype (37). Finally, the model showed high sensitivity for HCM screening, at the cost of a high false-positive rate. Expectedly, once a high probability of HCM is identified by an initial assessment, further examinations should be suggested to exclude HCM, as well as other common cardiovascular diseases, such as coronary heart disease. Therefore, this model may have practical value for further clinical application.

## Conclusion

This pragmatic model using only TWI and SV1 may be helpful to predict the probability of HCM and shows promise for use in population-based HCM screening.

## Data Availability Statement

The original contributions presented in the study are included in the article/Supplementary Material, further inquiries can be directed to the corresponding author/s.

## Ethics Statement

The studies involving human participants were reviewed and approved by the Medical Ethics Committee of the First Affiliated Hospital of the Air Force Medical University, and the requirement for written informed consent for this analysis was waived by the institutional review board.

## Author Contributions

LG, LL, FZ, and LT designed the study. LG, WY, ZM, MZ, HZ, GH, LZ, ZC, FS, and DS collected the data. LG, WY, ZM, JL, HS, BW, XG, and CH analyzed the data. LG and CG wrote the manuscript. LG, CG, FZ, LL, and LT revised the manuscript. LT provided fund assistance for the manuscript. All authors contributed to the article and approved the submitted version.

## Conflict of Interest

The authors declare that the research was conducted in the absence of any commercial or financial relationships that could be construed as a potential conflict of interest.

## Publisher’s Note

All claims expressed in this article are solely those of the authors and do not necessarily represent those of their affiliated organizations, or those of the publisher, the editors and the reviewers. Any product that may be evaluated in this article, or claim that may be made by its manufacturer, is not guaranteed or endorsed by the publisher.

## Footnotes

1
http://121.36.159.143:9999/hcm.do

## Abbreviations

HCM: hypertrophic cardiomyopathy
SCD: sudden cardiac death
AF: atrial fibrillation
BBB: bundle branch block
ECG: electrocardiogram
Echo: echocardiography
LVEF: left ventricular ejection fraction
LA: left atrium
SAM: systolic anterior motion
MWT: maximum wall thickness
LVH: left ventricular hypertrophy
TWI: T wave inversion
SV1: the S wave amplitude of lead V1
LASSO: the least absolute shrinkage and selection operator
LR: logistic regression
AIC: Akaike Information Criterion
BIC: Bayesian information criterion
ROC: receiver operator characteristic
R^2^: R square
CI: confidence interval.
