# Supplementary material for: Derivation and Validation of a Screening Model for Hypertrophic Cardiomyopathy Based on Electrocardiogram Features
Source: Front Cardiovasc Med. 2022 May 24;9:889523. doi: 10.3389/fcvm.2022.889523 (PMC9170889; doi:10.3389/fcvm.2022.889523)

**Supplementary tables**

**Supplementary Table 1. Checklist of TRIPOD items**

**Supplementary Table 2. ECG parameters**

Data are expressed as n (%) or median (IQR), unless otherwise specified.

TWI, T wave inversion; RⅠ, amplitude of R wave in lead Ⅰ; RV5SV1, amplitude of R wave in V5 plus S wave amplitude in V1; RISIII, amplitude of R wave in lead I plus S wave amplitude in lead III; RaVLSV3, amplitude of R wave in lead aVL plus S wave amplitude in precordial lead V3; RV1V2, amplitude of R wave in precordial lead V1 plus V2; SV1V2, amplitude of S wave in precordial lead V1 plus V2; RV2V3, amplitude of R wave in precordial lead V2 plus V3; SV2V3, amplitude of S wave in precordial lead V2 plus V3; RV3V4, amplitude of R wave in precordial lead V3 plus V4; SV3V4, amplitude of R wave in precordial lead V3 plus V4.

**Supplementary Table 3. LASSO analysis**

**Supplementary Table 4. Multivariable logistic regression with backward stepwise selection based on the minimum AIC**

**Supplementary Table 5. LASSO followed by multivariable logistic regression with backward stepwise selection based on the minimum AIC**

**Supplementary Table 6. LASSO followed by best subset selection based on the minimum AIC**

**Supplementary Table 7. Comparison of C-statistics between the two-variable models in the training, temporal validation, and external validation cohorts**

**Supplementary figures and figure legends**

**Supplementary Figure 1. Chart of C-statistic trend of distinct models with different numbers of variables in the temporal validation cohort**

**Supplementary Figure 2. ROC curve of the two-variable models for HCM screening in the training, temporal validation, and external validation cohorts**

**Supplementary Figure 3. ROC curve in the training cohort**

**Supplementary Table 1. Checklist of TRIPOD items**

| **Section/Topic** | **Item** | **Development or Validation?** | | **Checklist Item** | **Y/N** |
| --- | --- | --- | --- | --- | --- |
| Title and abstract |  | |  |  |  |
| Title | 1 | | D; V | Identify the study as developing and/or validating a multivariable prediction model, the target population, and the outcome to be predicted | Y |
| Abstract | 2 | | D; V | Provide a summary of objectives, study design, setting, participants, sample size, predictors, outcome, statistical analysis, results, and conclusions | Y |
| Introduction |  | |  |  |  |
| Background and objectives | 3a | | D; V | Explain the medical context (including whether diagnostic or prognostic) and rationale for developing or validating the multivariable prediction model, including references to existing models | Y |
|  | 3b | | D; V | Specify the objectives, including whether the study describes the development or validation of the model or both | Y |
| Methods |  | |  |  |  |
| Source of data | 4a | | D; V | Describe the study design or source of data (e.g., randomized trial, cohort, or registry data), separately for the development and validation data sets, if applicable | Y |
|  | 4b | | D; V | Specify the key study dates, including start of accrual; end of accrual; and, if applicable, end of follow-up | Y |
| Participants | 5a | | D; V | Specify key elements of the study setting (e.g., primary care, secondary care, general population) including number and location of center | Y |
|  | 5b | | D; V | Describe eligibility criteria for participants | Y |
|  | 5c | | D; V | Give details of treatments received, if relevant | N |
| Outcomes | 6a | | D; V | Clearly define the outcome that is predicted by the prediction model, including how and when assessed | Y |
|  | 6b | | D; V | Report any actions to blind assessment of the outcome to be predicted | Y |
| Predictors | 7a | | D; V | Clearly define all predictors used in developing the multivariable prediction model, including how and when they were measured | Y |
|  | 7b | | D; V | Report any actions to blind assessment of predictors for the outcome and other predictors | N |
| Sample size | 8 | | D; V | Explain how the study size was arrived at | Y |
| Missing data | 9 | | D; V | Describe how missing data were handled (e.g., complete-case analysis, single imputation, multiple imputation) with details of any imputation method | Y |
| Statistical analysis methods | 10a | | D | Describe how predictors were handled in the analyses | Y |
|  | 10b | | D | Specify type of model, all model-building procedures (including any predictor selection), and method for internal validation | Y |
|  | 10c | | V | For validation, describe how the predictions were calculated | Y |
|  | 10d | | D; V | Specify all measures used to assess model performance and, if relevant, to compare multiple models | Y |
|  | 10e | | V | Describe any model updating (e.g., recalibration) arising from the validation, if done | N |
| Risk groups | 11 | | D; V | Provide details on how risk groups were created, if done | Y |
| Development vs. validation | 12 | | V | For validation, identify any differences from the development data in setting, eligibility criteria, outcome, and predictors | Y |
| Results |  | |  |  |  |
| Participants | 13a | | D; V | Describe the flow of participants through the study, including the number of participants with and without the outcome and, if applicable, a summary of the follow-up time. A diagram may be helpful | Y |
|  | 13b | | D; V | Describe the characteristics of the participants (basic demographics, clinical features, available predictors), including the number of participants with missing data for predictors and outcome | Y |
|  | 13c | | V | For validation, show a comparison with the development data of the distribution of important variables (demographics, predictors and outcome) | Y |
| Model development | 14a | | D | Specify the number of participants and outcome events in each analysis | Y |
|  | 14b | | D | If done, report the unadjusted association between each candidate predictor and outcome | N |
| Model specification | 15a | | D | Present the full prediction model to allow predictions for individuals (i.e., all regression coefficients, and model intercept or baseline survival at a given time point) | Y |
|  | 15b | | D | Explain how to use the prediction model | Y |
| Model performance | 16 | | D; V | Report performance measures (with CIs) for the prediction model | Y |
| Model updating | 17 | | V | If done, report the results from any model updating (i.e., model specification, model performance) | N |
| Discussion |  | |  |  |  |
| Limitations | 18 | | D; V | Discuss any limitations of the study (such as nonrepresentative sample, few events per predictor, missing data) | Y |
| Interpretation | 19a | | V | For validation, discuss the results with reference to performance in the development data, and any other validation data | Y |
|  | 19b | | D; V | Give an overall interpretation of the results, considering objectives, limitations, results from similar studies, and other relevant evidence | Y |
| Implications | 20 | | D; V | Discuss the potential clinical use of the model and implications for future research | Y |
| Other information |  | |  |  |  |
| Supplementary information | 21 | | D; V | Provide information about the availability of supplementary resources, such as study protocol, Web calculator, and data sets | Y |
| Funding | 22 | | D; V | Give the source of funding and the role of the funders for the present study | Y |

**Supplementary Table 2. ECG parameters**

|  | **Overall** | | | **Training cohort** | | | | **Temporal internal validation cohort** | | | |
| --- | --- | --- | --- | --- | --- | --- | --- | --- | --- | --- | --- |
|  | **Non-HCM** | **HCM** | ***P*** | **Non-HCM** | **HCM** | ***P*** | **Non-HCM** | | **HCM** | ***P*** |  |
|  | **N=352** | **N=234** |  | **N=251** | **N=172** |  | **N=101** | | **N=62** |  |  |
| P, ms | 96 (88, 100) | 104 (98, 110) | <0.001 | 96 (88, 100) | 104 (97, 108) | <0.001 | 92 (85, 100) | | 105 (100, 114) | <0.001 |  |
| QRS, ms | 92 (84, 100) | 103 (92, 112) | <0.001 | 92 (84, 100) | 104 (92, 112) | <0.001 | 96 (82, 96) | | 100 (96, 108) | <0.001 |  |
| PR, ms | 148 (136, 162) | 152 (136, 168) | 0.036 | 148 (136, 164) | 152 (136, 168) | 0.370 | 147 (24) | | 154 (19) | 0.037 |  |
| QTc, ms | 409 (394, 425) | 428 (412, 443) | <0.001 | 410 (394, 425) | 428 (411, 440) | <0.001 | 406 (395, 426) | | 430 (412, 445) | <0.001 |  |
| Abnormal Q | 65 (18.5) | 56 (23.9) | 0.109 | 44 (17.5) | 36 (20.9） | 0.380 | 21 (20.8) | | 20 (32.3) | 0.101 |  |
| TWI | 33 (9.4) | 141 (60.3) | <0.001 | 25 (10.0) | 103 (59.9) | <0.001 | 8 (7.9) | | 38 (61.3) | <0.001 |  |
| RI, mv | 0.50 (0.30, 0.70) | 1.00 (0.62, 1.42) | <0.001 | 0.50 (0.30, 0.80) | 1.10 (0.67, 1.50) | <0.001 | 0.50 (0.39, 0.70) | | 0.92 (0.60, 1.33) | <0.001 |  |
| RaVL, mv | 0.20 (0.10, 0.40) | 0.60 (0.30, 1.03) | <0.001 | 0.20 (0.10, 0.40) | 0.60 (0.30, 1.10) | <0.001 | 0.20 (0.10, 0.40) | | 0.50 (0.29, 1.00) | <0.001 |  |
| RV1, mv | 0.20 (0.10, 0.40) | 0.40 (0.17, 0.80) | <0.001 | 0.20 (0.10, 0.40) | 0.39 (0.15, 0.80) | <0.001 | 0.20 (0.10, 0.45) | | 0.40 (0.20,0.90) | 0.001 |  |
| RV2, mv | 0.60 (0.34, 0.90) | 0.89 (0.44, 1.60) | <0.001 | 0.60 (0.39, 0.82) | 0.80 (0.33, 1.50) | <0.001 | 0.60 (0.30, 1.00) | | 1.08 (0.59,1.94) | <0.001 |  |
| RV3, mv | 0.90 (0.60, 1.40) | 1.65 (0.90, 2.56) | <0.001 | 0.90 (0.60, 1.35) | 1.50 (0.80, 2.57) | <0.001 | 1.03 (0.50, 1.40) | | 2.10 (1.10,2.59) | <0.001 |  |
| RV4, mv | 1.40 (1.00, 1.90) | 2.50 (1.50, 3.60) | <0.001 | 1.40 (1.10, 1.90) | 2.50 (1.53, 3.59) | <0.001 | 1.40 (1.00, 1.90) | | 2.53 (1.50, 3.63) | <0.001 |  |
| RV5, mv | 1.40 (1.03, 1.80) | 2.60 (1.50, 3.50) | <0.001 | 1.40 (1.06, 1.80) | 2.60 (1.50, 3.50) | <0.001 | 1.40 (1.00, 1.88) | | 2.42 (1.49, 3.27) | <0.001 |  |
| RV6, mv | 1.10 (0.80, 1.50) | 2.00 (1.29, 2.80) | <0.001 | 1.20 (0.80, 1.50) | 1.90 (1.20, 2.80) | <0.001 | 1.08 (0.80, 1.40) | | 2.05 (1.30, 2.80) | <0.001 |  |
| SIII, mv | 0.05 (0.00, 0.29) | 0.30 (0.00, 0.72) | <0.001 | 0.10 (0.00, 0.30) | 0.30 (0.00, 0.70) | <0.001 | 0.00 (0.00, 0.29) | | 0.30 (0.00, 0.79) | <0.001 |  |
| SV1, mv | 0.80 (0.60, 1.20) | 1.70 (1.08, 2.40) | <0.001 | 0.80 (0.60, 1.20) | 1.70 (1.00, 2.50) | <0.001 | 0.80 (0.53, 1.20) | | 1.51 (1.08, 2.30) | <0.001 |  |
| SV2, mv | 1.30 (0.90, 1.77) | 2.10 (1.30, 2.76) | <0.001 | 1.30 (0.90, 1.70) | 2.10 (1.30, 2.86) | <0.001 | 1.30 (0.92, 1.80) | | 2.20 (1.40, 2.56) | <0.001 |  |
| SV3, mv | 0.90 (0.56, 1.30) | 1.50 (0.73, 2.40) | <0.001 | 0.90 (0.57, 1.30) | 1.50 (0.71, 2.39) | <0.001 | 0.81 (0.55, 1.35) | | 1.50 (0.78, 2.41) | <0.001 |  |
| SV4, mv | 0.60 (0.30, 0.90) | 0.90 (0.48, 1.80) | <0.001 | 0.60 (0.30, 0.90) | 0.98 (0.42, 1.80) | <0.001 | 0.60 (0.40, 0.93) | | 0.90 (0.50, 1.93) | 0.001 |  |
| SV5, mv | 0.30 (0.16, 0.90) | 0.50 (0.20, 1.00) | <0.001 | 0.30 (0.15, 0.50) | 0.49 (0.20, 1.00) | <0.001 | 0.40 (0.20, 0.50) | | 0.50 (0.29, 1.13) | 0.014 |  |
| SV6, mv | 0.10 (0.00, 0.30) | 0.20 (0.00, 0.50) | <0.001 | 0.10 (0.00, 0.30) | 0.20 (0.00, 0.43) | 0.019 | 0.15 (0.00, 0.30) | | 0.30 (0.10, 0.56) | 0.004 |  |
| RV5SV1, mv | 2.30 (1.80, 2.90) | 4.15 (3.07, 5.80) | <0.001 | 2.30 (1.80, 2.90) | 4.16 (3.10, 5.90) | <0.001 | 2.40 (1.68, 2.90) | | 4.15 (2.92, 5.43) | <0.001 |  |
| RISIII, mv | 0.60 (0.40, 0.93) | 1.40 (0.80, 2.20) | <0.001 | 0.60 (0.40, 1.00) | 1.43 (0.80, 2.20) | <0.001 | 0.60 (0.44, 0.90) | | 1.30 (0.90, 2.10) | <0.001 |  |
| RaVLSV3, mv | 1.13 (0.79, 1.70) | 2.30 (1.30, 3.04) | <0.001 | 1.20 (0.80, 1.70) | 2.30 (1.40, 3.00) | <0.001 | 1.10 (0.74, 1.65) | | 2.23 (1.30, 3.10) | <0.001 |  |
| RV1V2, mv | 0.80 (0.50, 1.30) | 1.30 (0.60, 2.40) | <0.001 | 0.80 (0.50, 1.30) | 1.30 (0.50, 2.29) | <0.001 | 0.90 (0.50, 1.42) | | 1.60 (0.76, 3.00) | <0.001 |  |
| SV1V2, mv | 2.01 (1.37, 2.78) | 3.76 (2.60, 4.97) | <0.001 | 2.15 (1.60, 2.86) | 3.80 (2.54, 5.20) | <0.001 | 1.50 (0.82, 2.28) | | 3.69 (2.70, 4.83) | <0.001 |  |
| RV2V3, mv | 1.50 (1.00, 2.21) | 2.60 (1.50, 4.21) | <0.001 | 1.50 (1.00, 2.20) | 2.45 (1.40, 4.10) | <0.001 | 1.60 (0.98, 2.40) | | 2.95 (2.08, 4.83) | <0.001 |  |
| SV2V3, mv | 2.10 (1.31, 2.98) | 3.70 (2.22, 4.85) | <0.001 | 2.20 (1.60, 3.00) | 3.70 (2.25, 4.88) | <0.001 | 1.60 (0.73, 2.43) | | 3.70 (2.18, 4.87) | <0.001 |  |
| RV3RV4, mv | 2.40 (1.70, 3.20) | 4.20 (2.50, 6.00) | <0.001 | 2.40 (1.80, 3.20) | 4.10 (2.50, 6.00) | <0.001 | 2.40 (1.50, 3.30) | | 4.55 (2.54, 6.15) | <0.001 |  |
| SV3SV4, mv | 1.50 (0.90, 2.28) | 2.50 (1.30, 4.24) | <0.001 | 1.50 (0.90, 2.20) | 2.60 (1.20, 4.20) | <0.001 | 1.41 (0.96, 2.36) | | 2.43 (1.34, 4.31) | <0.001 |  |

Data are expressed as n (%) or median (IQR), unless otherwise specified.

TWI, T wave inversion; RⅠ, amplitude of R wave in lead Ⅰ; RV5SV1, amplitude of R wave in V5 plus S wave amplitude in V1; RISIII, amplitude of R wave in lead I plus S wave amplitude in lead III; RaVLSV3, amplitude of R wave in lead aVL plus S wave amplitude in precordial lead V3; RV1V2, amplitude of R wave in precordial lead V1 plus V2; SV1V2, amplitude of S wave in precordial lead V1 plus V2; RV2V3, amplitude of R wave in precordial lead V2 plus V3; SV2V3, amplitude of S wave in precordial lead V2 plus V3; RV3V4, amplitude of R wave in precordial lead V3 plus V4; SV3V4, amplitude of R wave in precordial lead V3 plus V4.

**Supplementary Table 3. LASSO analysis**

| **Models** | **Number of variables** | **AIC** | **BIC** | **R^2^** | **C-statistic**  **(training)** | **C-statistic**  **(validation)** |
| --- | --- | --- | --- | --- | --- | --- |
| P+QRS+TWI+QTc+RV1+RV3+SV1+SV2+SV4+SV6+SⅢ+ RV3V4 | 12 | 322.709 | 374.435 | 0.646 | 0.931 | 0.931 |
| P+QRS+TWI+QTc+RV1+RV3+SV1+SV4+SV6+SⅢ+ RV3V4 | 11 | 321.701 | 369.508 | 0.644 | 0.929 | 0.939 |
| P+QRS+TWI+QTc+RV1+RV3+SV1+SV4+SV6+SⅢ+RⅠSⅢ+RV3V4 | 10 | 365.044 | 408.922 | 0.564 | 0.931 | 0.936 |
| P+QRS+TWI+QTc+SV1+SV4+RV5SV1+RⅠSⅢ+RV2V3 | 9 | 396.730 | 436.670 | 0.499 | 0.924 | 0.934 |
| TWI+RV5SV1+QRS+RⅠSⅢ+P+SV1+RV2V3+SV4 | 8 | 416.730 | 452.721 | 0.454 | 0.916 | 0.928 |
| TWI+RV5SV1+QRS+RⅠSⅢ+P+SV1+RV2V3 | 7 | 420.114 | 452.145 | 0.442 | 0.912 | 0.930 |
| TWI+RV5SV1+QRS+RⅠSⅢ+P+SV1 | 6 | 420.640 | 448.702 | 0.436 | 0.911 | 0.923 |
| TWI+RV5SV1+QRS+RⅠSⅢ+P | 5 | 420.995 | 445.077 | 0.431 | 0.907 | 0.921 |
| TWI+RV5SV1+QRS+RⅠSⅢ | 4 | 423.498 | 443.591 | 0.421 | 0.891 | 0.913 |
| TWI+RV5SV1+QRS | 3 | 429.634 | 445.728 | 0.403 | 0.882 | 0.895 |
| TWI+RV5SV1 | 2 | 449.023 | 461.108 | 0.354 | 0.862 | 0.871 |

**Supplementary Table 4. Multivariable logistic regression with backward stepwise selection based on the minimum AIC**

| **Models** | **Number of variables** | **AIC** | **BIC** | **R^2^** | **C-statistic**  **(training)** | **C-statistic**  **(validation)** |
| --- | --- | --- | --- | --- | --- | --- |
| P+QRS+TWI+QTc+SV1+SV2+SV4+SV5+SV6+SⅢ+RaVLSV3+ RV1V2+RV3V4 | 13 | 307.448 | 363.082 | 0.513 | 0.935 | 0.929 |
| P+QRS+TWI+QTc+SV1+ SV4+SV5+SV6+SⅢ+RaVLSV3+ RV1V2+RV3V4 | 12 | 310.585 | 362.311 | 0.504 | 0.934 | 0.935 |
| P+QRS+TWI+QTc+SV1+ SV4+SV5+SV6+SⅢ+ RaVLSV3+RV3V4 | 11 | 311.231 | 359.039 | 0.499 | 0.932 | 0.932 |
| P+QRS+TWI+QTc+SV1+SV4+SV5+SⅢ+RaVLSV3+RV3V4 | 10 | 313.922 | 357.800 | 0.490 | 0.932 | 0.928 |
| P+QRS+TWI+QTc+SV1+SV4+SⅢ+RaVLSV3+RV3V4 | 9 | 313.068 | 353.008 | 0.488 | 0.932 | 0.934 |
| P+QRS+TWI+QTc+SV1+SV4+SⅢ+RV3V4 | 8 | 317.749 | 353.740 | 0.476 | 0.928 | 0.935 |
| P+QRS+TWI+QTc+SV1+SⅢ+RV3V4 | 7 | 316.616 | 348.647 | 0.475 | 0.926 | 0.937 |
| P+QRS+TWI+QTc+SV1+RV3V4 | 6 | 322.152 | 350.214 | 0.461 | 0.919 | 0.925 |
| P+QRS+TWI+ SV1+ RV3V4 | 5 | 332.638 | 356.720 | 0.439 | 0.908 | 0.920 |
| QRS+TWI+ SV1+ RV3V4 | 4 | 349.072 | 369.165 | 0.407 | 0.893 | 0.912 |
| QRS+TWI+ SV1 | 3 | 366.631 | 382.725 | 0.373 | 0.881 | 0.889 |
| TWI+ SV1 | 2 | 393.220 | 405.305 | 0.323 | 0.857 | 0.868 |

**Supplementary Table 5. LASSO followed by multivariable logistic regression with backward stepwise selection based on the minimum AIC**

| **Models** | **Number of variables** | **AIC** | **BIC** | **R^2^** | **C-statistic**  **(training)** | **C-statistic**  **(validation)** |
| --- | --- | --- | --- | --- | --- | --- |
| P+QRS+TWI+QTc+RV1+RV3+SV1+SV2+SV4+SV6+SⅢ+ RV3V4 | 12 | 313.126 | 364.851 | 0.499 | 0.931 | 0.931 |
| P+QRS+TWI+QTc+RV1+RV3+SV1+SV2+SV4+SV6+SⅢ | 11 | 311.508 | 359.315 | 0.498 | 0.931 | 0.929 |
| P+QRS+TWI+QTc+RV1+RV3+SV1+SV2+SV4+ SⅢ | 10 | 310.468 | 354.346 | 0.496 | 0.929 | 0.928 |
| P+SV1+SV2+RV3+RV1+QTc+TWI+QRS+SV4 | 9 | 310.423 | 350.363 | 0.493 | 0.927 | 0.923 |
| P+SV1+SV2+RV3+QTc+TWI+QRS+SV4 | 8 | 310.933 | 346.923 | 0.488 | 0.925 | 0.917 |
| P+SV1+SV2+RV3+QTc+TWI+SV4 | 7 | 317.595 | 349.627 | 0.473 | 0.922 | 0.911 |
| P+SV1+RV3+QTc+TWI+SV4 | 6 | 324.917 | 352.979 | 0.457 | 0.919 | 0.923 |
| P+SV1+RV3+QTc+TWI | 5 | 331.641 | 355.724 | 0.441 | 0.909 | 0.920 |
| P+SV1+RV3+TWI | 4 | 347.344 | 367.437 | 0.410 | 0.896 | 0.910 |
| SV1+RV3+TWI | 3 | 366.637 | 382.731 | 0.373 | 0.878 | 0.901 |
| SV1+TWI | 2 | 393.220 | 405.305 | 0.323 | 0.857 | 0.868 |

**Supplementary Table 6. LASSO followed by best subset selection based on the minimum AIC**

| **Models** | **Number of variables** | **AIC** | **BIC** | **R^2^** | **C-statistic**  **(training)** | **C-statistic**  **(validation)** |
| --- | --- | --- | --- | --- | --- | --- |
| P+SV4+SV1+SV2+RV3+RV1+TWI+QTc+QRS | 9 | 310.423 | 350.363 | 0.656 | 0.927 | 0.923 |
| P+SV4+SV1+SV2+RV3+TWI+QTc+QRS | 8 | 310.933 | 346.923 | 0.652 | 0.925 | 0.917 |
| P+SV1+RV3+TWI+QTc+QRS+SV6 | 7 | 315.087 | 347.119 | 0.641 | 0.923 | 0.929 |
| P+SV1+RV3+TWI+QTc+SV6 | 6 | 319.504 | 347.566 | 0.630 | 0.919 | 0.928 |
| P+SV1+RV3+TWI+SV6 | 5 | 330.829 | 354.911 | 0.607 | 0.909 | 0.920 |
| SV1+TWI+SV6+RV3V4 | 4 | 346.323 | 366.415 | 0.576 | 0.894 | 0.908 |
| SV1+TWI+RV3V4 | 3 | 366.561 | 382.654 | 0.534 | 0.878 | 0.899 |
| TWI+SV1 | 2 | 393.220 | 405.305 | 0.477 | 0.857 | 0.868 |

| **Methods** | **Models** | **C-statistic (95% CI) (Training cohort)** | ***P* value**  **(Delong test)** | **C-statistic (95% CI)**  **(Temporal validation cohort)** | ***P* value**  **(Delong test)** | **C-statistic (95% CI）**  **(External validation cohort)** | ***P* value**  **DeLong test** |
| --- | --- | --- | --- | --- | --- | --- | --- |
| Backward stepwise LR;  LASSO + backward stepwise LR;  LASSO+best subset | TWI+SV1 | 0.857 (0.818-0.896) | - | 0.871 (0.812-0.930) | - | 0.833 (0.825-0.841) | - |
| LASSO | TWI+RV5SV1 | 0.862 (0.824-0.901) | 0.649 | 0.872 (0.812-0.932) | 0.942 | 0.808 (0.798-0.819) | <0.001 |

**Supplementary Table 7. Comparison of C-statistics between the two-variable models in the training, temporal validation, and external validation cohorts**

**Supplementary figures and figure legends**

**Supplementary Figure 1. Chart of C-statistic trend of distinct models with different numbers of variables in the temporal validation cohort**

**Supplementary Figure 2. ROC curve of the two-variable models for HCM screening in the training, temporal validation, and external validation cohorts**

**Supplementary Figure 3. ROC curve in the training cohort**

**Supplementary Figure 1. Tendency chart of C-statistics of distinct models for different numbers of variable combinations** **in the temporal validation cohort**


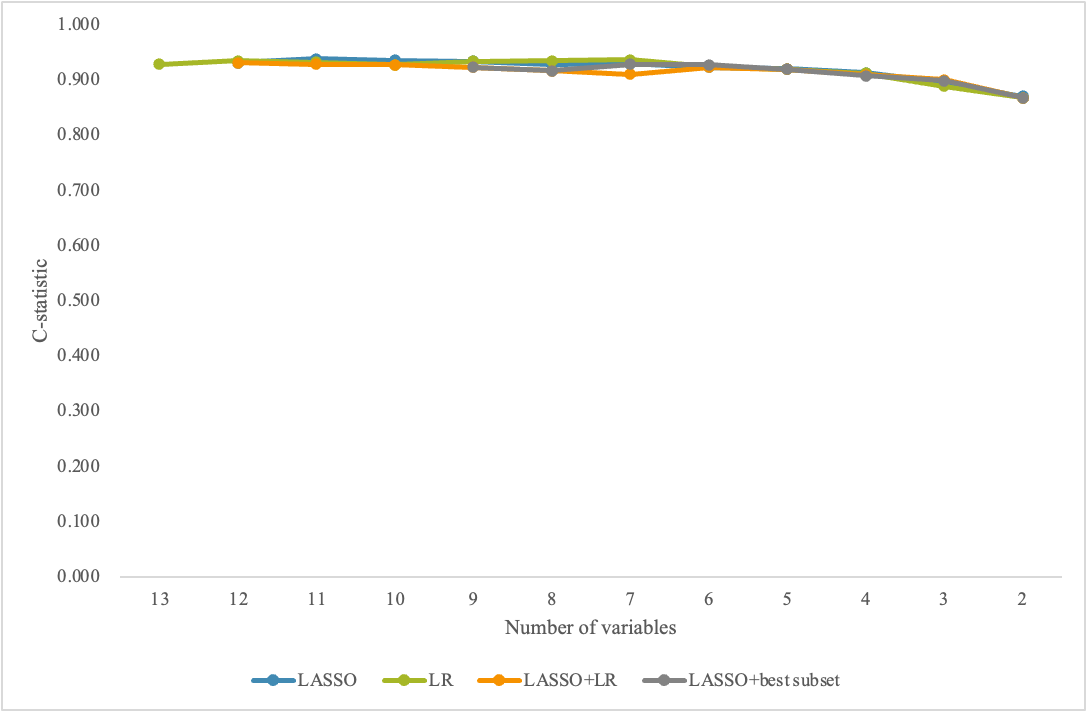

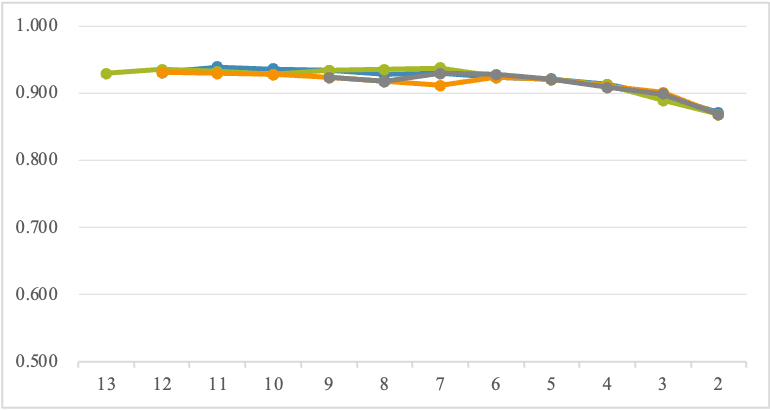


**Supplementary Figure 2. ROC curve of the two variables models for screening HCM in the training, temporal validation, and external validation cohorts**


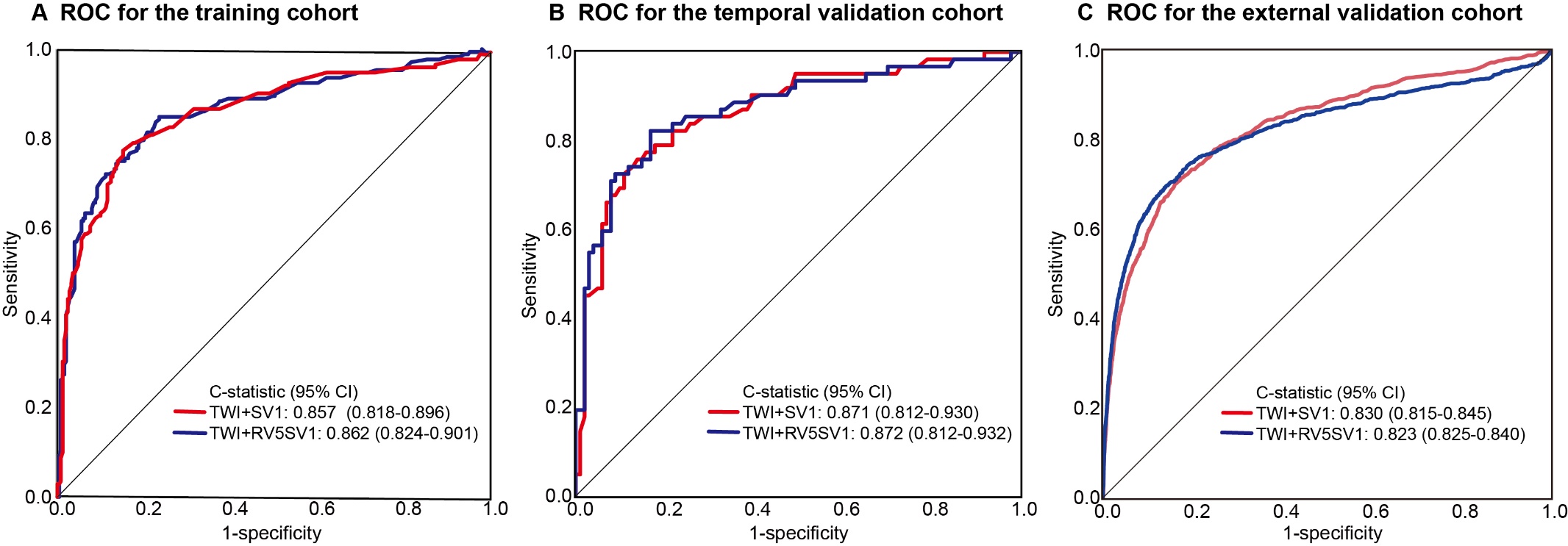


**Supplementary Figure 3. ROC curve in the training cohort**


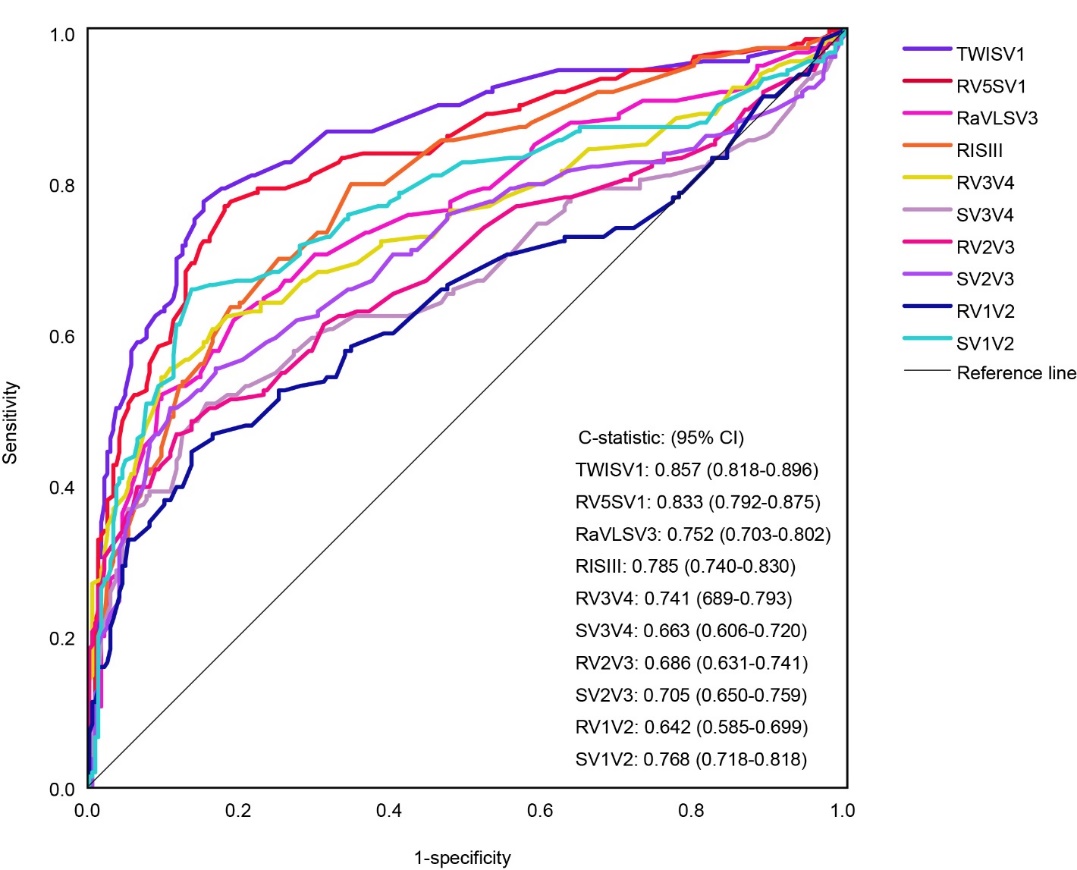

Supplement: Supplementary file 1 [file Data_Sheet_1.docx]
